# Supplementary material for: Identification of photoperiod-induced specific miRNAs in the adrenal glands of Sunite sheep (Ovis aries)
Source: Front Vet Sci. 2022 Jul 22;9:888207. doi: 10.3389/fvets.2022.888207 (PMC9354845; doi:10.3389/fvets.2022.888207)
Supplement: Supplementary file 1 [file Data_Sheet_1.ZIP › Supplementary file/miRDeep2 file.zip!/miRDeep2 file/Novel_Structure/LP42A3/Novel_111.pdf]

Provisional ID	:	NC_019458.2_591
Score total	:	0.8
Score for star read(s)	:	-1.3
Score for read counts	:	0
Score for mfe	:	0.5
Score for randfold	:	1.6
Score for cons. seed	:	
Total read count	:	157
Mature read count	:	157
Loop read count	:	0
Star read count	:	0

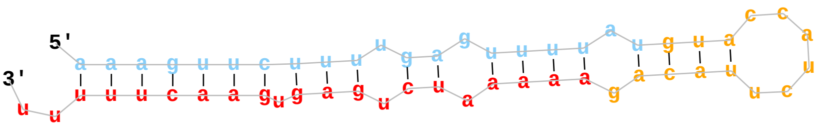

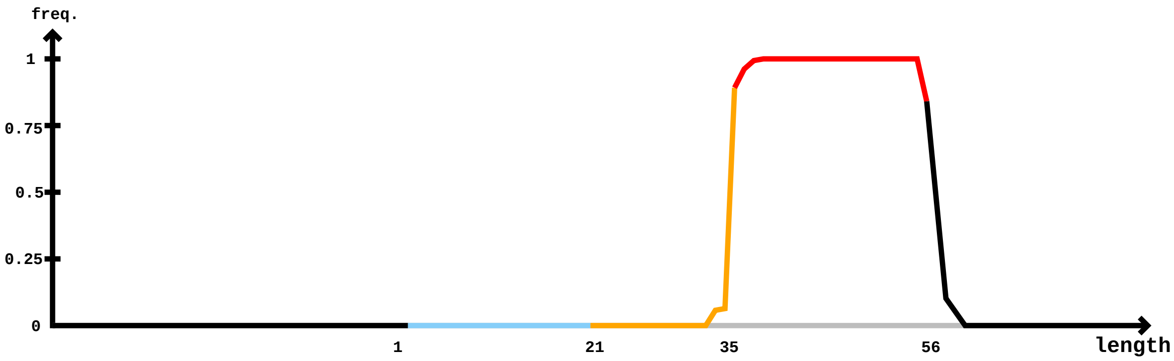

Star **Mature**

```
5'- auauuuuuauuuguaacuaaguaugggcugaaaaaaaaguucuuuugaguuuuauguaccaucuuacagaaaaaucugagugaacuuuuuggccagcccaguauuaaauaca  -3'   exp
    ..........((((..(((...(((((((...(((((((((((.((.((((.((((......)))).)))).)).))).)))))))))...)))))))...)))..))))   reads   mm   sample
    ...................................................................agaaaaaucugaAugaacuuuu.......................   2       1    seq
    ...................................................................agaaaaaucugaAugaacuuuuu......................   7       1    seq
    ....................................................................gaaaaaucugaAugaacuuuuu......................   1       1    seq
    .....................................................................aaaaaucugaAugaacuuuu.......................   23      1    seq
    .....................................................................aaaaaucugaAugaacuuuuu......................   65      1    seq
    .....................................................................aaaaaucugaAugaacuuuuug.....................   42      1    seq
    ......................................................................aaaaucugagugaacuuuuu......................   1       0    seq
    ......................................................................aaaaucugagugaacuuuuugg....................   8       0    seq
    ......................................................................aaaaucugaAugaacuuuuuggc...................   2       1    seq
    .......................................................................aaaucugagugaacuuuuuggc...................   5       0    seq
    ........................................................................aaucugagugaacuuuuuggc...................   1       0    seq
```
